# Acute effects of (–)-gallocatechin gallate-rich green tea extract on the cerebral hemodynamic response of the prefrontal cortex in healthy humans

**DOI:** 10.3389/fnrgo.2023.1136362

**Published:** 2023-11-27

**Authors:** Jihyun Cha, Hyung-Su Kim, Gusang Kwon, Si-Young Cho, Jae-Myoung Kim

**Affiliations:** ^1^Department of Research and Development, OBELAB Inc., Seoul, Republic of Korea; ^2^Amorepacific R&I Center, Yongin-si, Republic of Korea

**Keywords:** gallocatechin gallate, green tea, hemodynamic response, cognitive function, neural efficiency, fNIRS

## Abstract

**Objective:**

The benefits of long-term consumption of green tea on the brain are well known. However, among many ingredients of green tea, the acute effects of (–)-gallocatechin gallate-rich green tea extract (GCG-GTE), have received comparatively less attention. Herein, we investigated the acute effects of oral ingestion of green tea with GCG-GTE, which contains close replicas of the ingredients of hot green tea, on task-dependent hemodynamics in the prefrontal cortex of healthy adult human brains.

**Methods:**

In this randomized, double-blind, placebo-controlled, parallel group trial, 35 healthy adults completed computerized cognitive tasks that demand activation of the prefrontal cortex at baseline and 1 h after consumption of placebo and 900 mg of GCG-GTE extract supplement. During cognitive testing, hemodynamic responses (change in HbO2 concentration) in the prefrontal cortex were assessed using functional near-infrared spectroscopy (fNIRS).

**Results:**

In fNIRS data, significant group x session interactions were found in the left (*p* = 0.035) and right (*p* = 0.036) dorsolateral prefrontal cortex (DLPFC). In behavioral data, despite the numerical increase in the GCG-GTE group and the numerical decrease in the Placebo group, no significant differences were observed in the cognitive performance measure between the groups.

**Conclusion:**

The result suggests a single dose of orally administered GCG-GTE can reduce DLPFC activation in healthy humans even with increased task demand. GCG-GTE is a promising functional material that can affect neural efficiency to lower mental workload during cognitively demanding tasks. However, further studies are needed to verify this.

## 1 Introduction

Green tea has been a popular beverage in Asia for thousands of years. In addition to its unique taste, green tea is consumed for its mental health benefits, such as prevention of neurodegenerative diseases, relaxation, mental clarity, and concentration (Dietz and Dekker, 2017). Epidemiological studies have shown that long-term consumption of green tea reduces the risk of cognitive impairment in the elderly (Feng et al., 2010; Ma et al., 2016). In addition to its long-term benefits, green tea consumption has shown immediate improvement in mental performance, particularly attentional function, in young adults with mild acute psychological stress (Baba et al., 2021).

Within the diverse components of tea, caffeine and theanine are implicated in the acute effects of green tea, such as mood regulation. In contrast, catechins are involved in the long-term effects of green tea, particularly in areas such as cognition (Einöther and Martens, 2013; Noguchi-Shinohara et al., 2014). Caffeine affects various neurotransmitters, including noradrenaline, acetylcholine, dopaminergic neurotransmitters, serotonin, and glutamate, leading to increased arousal, alertness, and improvement of executive functions and attention (Fredholm et al., 1999; Bryan, 2008). In contrast, theanine, a unique amino acid found in tea, reduces anxiety and induces relaxation or calm by attenuating neurotransmitter levels or increasing alpha-band activity (Fredholm et al., 1999; Lu et al., 2004).

Tea catechins, one of the main beneficial components of green tea, are classified as epi-type and epimer-type catechins. Epi-type catechins are (–)-epigallocatechin-3-gallate (EGCG), (–)-epigallocatechin (EGC), (–)-epicatechin-3-gallate (ECG), and (–)-epicatechin (EC), which are prominently present in green tea leaves. Epimer-type catechins are the stereochemical isomers of epi-type catechins, including (–)-gallocatechin gallate (GCG), (–)-catechin gallate (CG), (–)-gallocatechin (GC), and (–)-catechin (C) (Seto et al., 1997). As epimer-type catechins are produced by heat treatment during tea manufacturing processes, they are rarely present in fresh or dried green tea leaves, but are plentiful in processed green tea products, such as canned or bottled tea drinks (Wang and Helliwell, 2000; Chen et al., 2001; Xu et al., 2003). In the actual tea beverage that we consume, epicatechin and epimer-type catechin are present in nearly equal proportions. For this reason, conducting research to validate the genuine effects of tea through substances that closely emulate the composition of actual tea holds considerable value.

EGCG, the most abundant epi-type catechin, contributes to the chronic mental health benefits of green tea (Mähler et al., 2013). Long-term consumption of EGCG exerts neuroprotective effects and ameliorates memory impairment through its antioxidant and anti-inflammatory properties as well as its ability to reduce Aβ levels (Weinreb et al., 2004; Ehrnhoefer et al., 2008; Singh et al., 2016). Compared to the many studies on the chronic effects of EGCG on cognition and health (Payne et al., 2022), there are limited studies on its acute impact on cognitive benefits. In an electroencephalogram (EEG) study, Scholey et al. demonstrated that the EGCG treatment group showed overall increases in alpha, beta and theta activity in the frontal and medial frontal gyrus. Together with self-ratings of increased calmness and reduced stress, the authors concluded that the participants may have been in a more relaxed and attentive states after consuming EGCG (Scholey et al., 2012). However, in a study using functional near-infrared spectroscopy (fNIRS), the EGCG administration group showed cerebral blood flow reduction in the frontal cortex, but no differences in cognitive performance and mood measures (Wightman et al., 2012).

In contrast, GCG, an epimer counterpart of EGCG, has a 2.4 times higher blood–brain barrier permeability than EGCG (Jeong et al., 2018). Furthermore, GCG has stronger scavenging ability and inhibition of Aβ aggregation than EGCG (Guo et al., 1999), suggesting that GCG contributes significantly to the cognitive health benefits of green tea consumption. Recently, several studies have been conducted using various animal models to verify the beneficial effects of GCG-rich green tea extracts (GCG-GTE) on cognition. One study revealed that long-term consumption of GCG-GTE attenuated scopolamine-induced cognitive impairment in mice (Bae et al., 2020). Another study reported that dietary supplementation of GCG-GTE for 10 weeks alleviated cognitive dysfunction in Alzheimer's disease model (Kim et al., 2019). The other study reported that oral administrations of GCG-GTE for 4 weeks rescued age-related cognitive deficits in the late middle-aged murine model (Ahn et al., 2022). Furthermore, in a double-blind, placebo-controlled, randomized clinical study on human subjects, GCG-GTE intake for 8 weeks had beneficial effects on cognitive function, particularly attention and working memory, in middle-aged healthy adults (Kwon et al., 2020). These results suggest that long-term intake of GCG-GTE may improve cognitive function, but the acute effect of the extract on cognition has not yet been evaluated.

In this study, we investigated the acute effects of GCG-GTE on task-dependent hemodynamic changes in prefrontal regions. We utilized a fNIRS system, which can measure regional cerebral hemodynamics in a non-invasive manner, to collect brain activity data. Focusing on capturing the behavioral and neuronal benefits of GCG-GTE on cognition, we administered three cognitively demanding tasks, each requiring intense working memory utilization and a high level of executive control. In addition, using a multichannel portable fNIRS device, we aimed to take a closer look at the key regions known to actively participate during cognitive tasks, namely, the bilateral dorsal lateral prefrontal cortex (DLPFC). To the best of our knowledge, this is the first report on the acute effects of GCG-rich green tea.

## 2 Materials and methods

### 2.1 Test substances

Fresh green tea (*Camellia sinensis*) leaves were collected in the spring from the Osulloc Tea Garden in Jeju, Korea and dried. Dried *C. sinensis* leaves were subjected to extraction twice with 50% aqueous ethanol and incubation at 100°C (1.2 atm) under aqueous conditions for 5 h to obtain GCG-GTE. The catechin contents in GCG-GTE were measured and analyzed using high-performance liquid chromatography (HPLC) with a photodiode array (PDA) detector (Alliance 2695 system, Waters) and a Thermo Syncronis C18 column (250 × 4.6 mm, I.D., 5 μm; Thermo Fisher Scientific Inc.) GCG-GTE contained almost the same amount of epicatechins and epicatechin epimers (Table 1).

**Table 1:** Chemical constituents of GCG-GTE.

**Component**		**Content (mg/g GCG-GTE)**
Catechin epimers	Catechin	14.8 ± 0.6
		Catechin gallate	13.2 ± 0.2
		Gallocatechin	46.4 ± 1.3
		Gallocatechin gallate	56.4 ± 0.4
*Sum of catechin epimers*		*130.8 ± 2.5*
Epicatechins	Epicatechin	9.2 ± 0.4
		Epicatechin gallate	13.0 ± 0.0
		Epigallocatechin	32.8 ± 1.0
		Epigallocatechin gallate	53.2 ± 0.5
*Sum of epicatechins*		*108.2 ± 1.9*
**Total catechins**		**239.6** **±0.8**
Caffeine		38.6 ± 0.5
L-theanine		11.2 ± 0.5

GCG-GTE, (–)-gallocatechin gallate-rich green tea extracts.

### 2.2 Study design and participants

**Figure 1:**
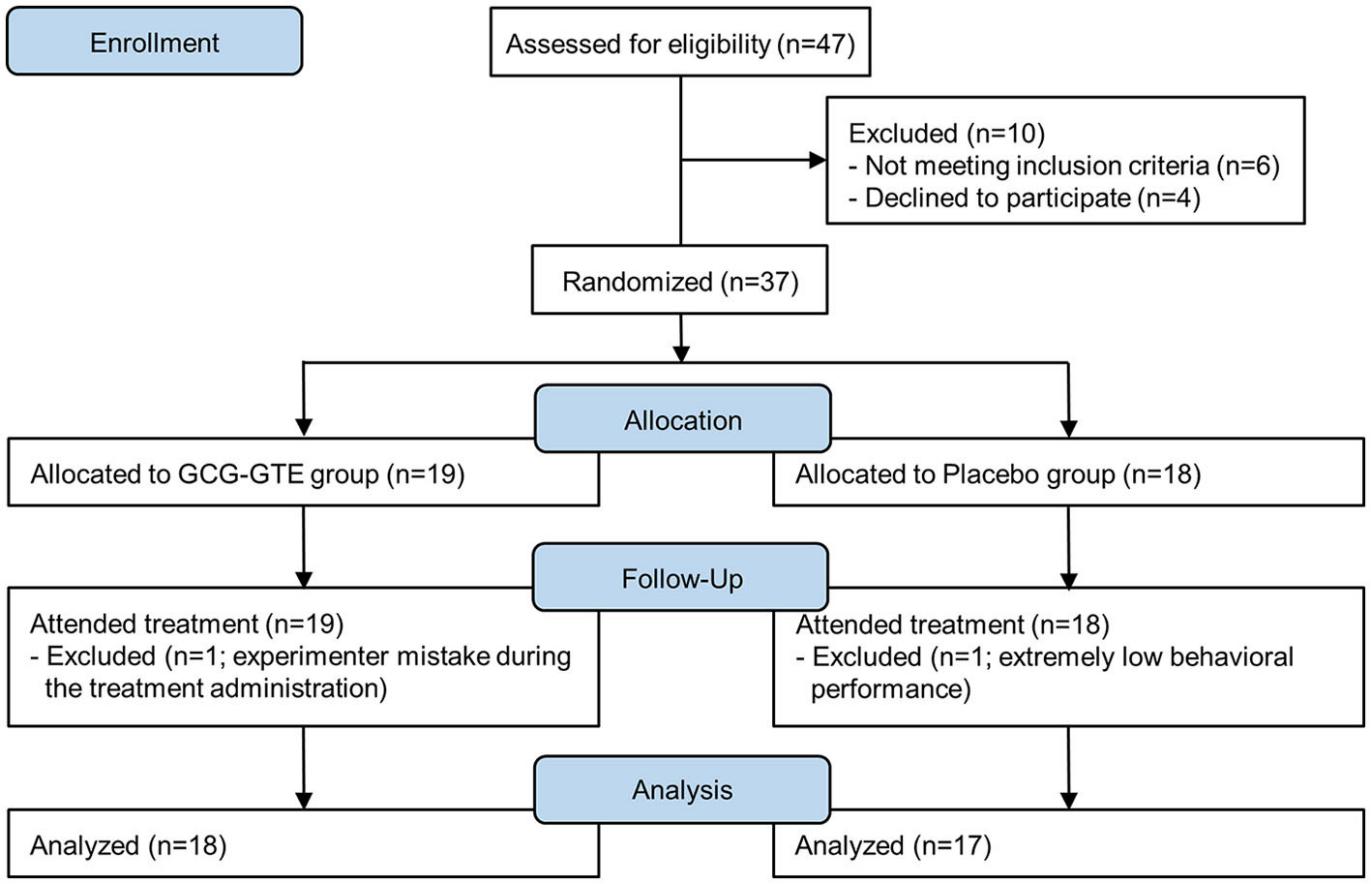
Study flow diagram.

Thirty-seven young adults [mean age = 32.4, standard deviation (SD) = 4.27, 16 females] were recruited from Amorepacific Inc. (Seoul, Republic of Korea) for participation in the study. The exclusion criteria for participants were as follows; (1) A person who has taken health functional foods related to improving memory and cognitive function within the previous month (2) A person who has consumed health functional foods extracted from green tea within the previous month or who has consumed more than four cups of green tea a day. Among the 37 participants, one participant was excluded due to extremely low behavioral performance (task score was less than three SDs from the average score), and another was excluded because of an experimental error during treatment administration. Therefore, 35 participants were included in the final analysis (Figure 1). None of the remaining participants had cognitive or other neurological disorders. All participants attended the Amorepacific consumer research center 1 h prior to testing without consuming caffeine or alcohol from 20:00 the night before their participation. Informed consent was obtained before the experiment according to the procedure approved by the Amorepacific R&I Institutional Review Board (IRB: 2019-1CR-N076S). This study was also registered with the Clinical Research Information Service (registration number: KCT0004808), Republic of Korea (URL link: https://cris.nih.go.kr/cris/index/index.do). Given the proprietary issues with GCG-GTE at the time of recruitment, this study was registered retrospectively.

### 2.3 Treatments

During the visit, the participants were randomly assigned to either the placebo or GCG-GTE group, where the order of allocation was counterbalanced via the Latin square method. The participants in each group were administered a single dose of four tablets. Each tablet for the placebo and GCG-GTE groups contained an inert placebo (225 mg of maltodextrin, predominately) and GCG-GTE (225 mg), respectively. The tablets for the groups were identical in size and color and were prepared and coded by a third party who had no further involvement in any aspect of the study. No member of the research team was aware of the contents of the tablets until a blind data review was completed.

### 2.4 Measurement procedure and cognitive tasks

For assessment of the differences in cerebral activation following the consumption of GCG-GTE, participants performed a series of computerized cognitive tasks—mental arithmetic (MA) task, Corsi block-tapping task (CBTT), and verbal fluency task (VFT)—after prior measurements in the 3-min resting state. This protocol was employed to induce lower or higher activation of the prefrontal cortex, as in a previous study (Wightman et al., 2012).

**Figure 2:**
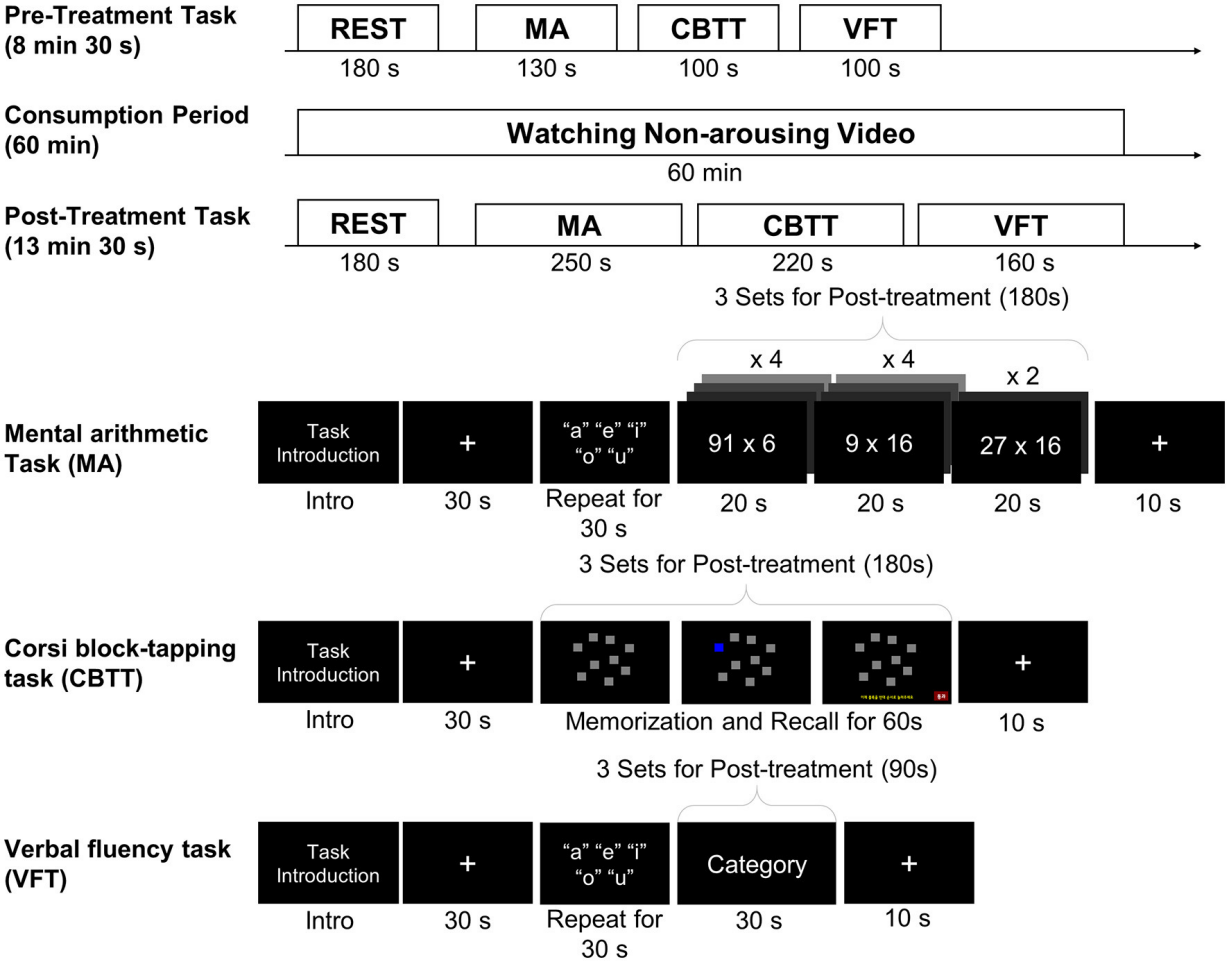
Schematic illustration of the experimental protocol.

**Figure 3:**
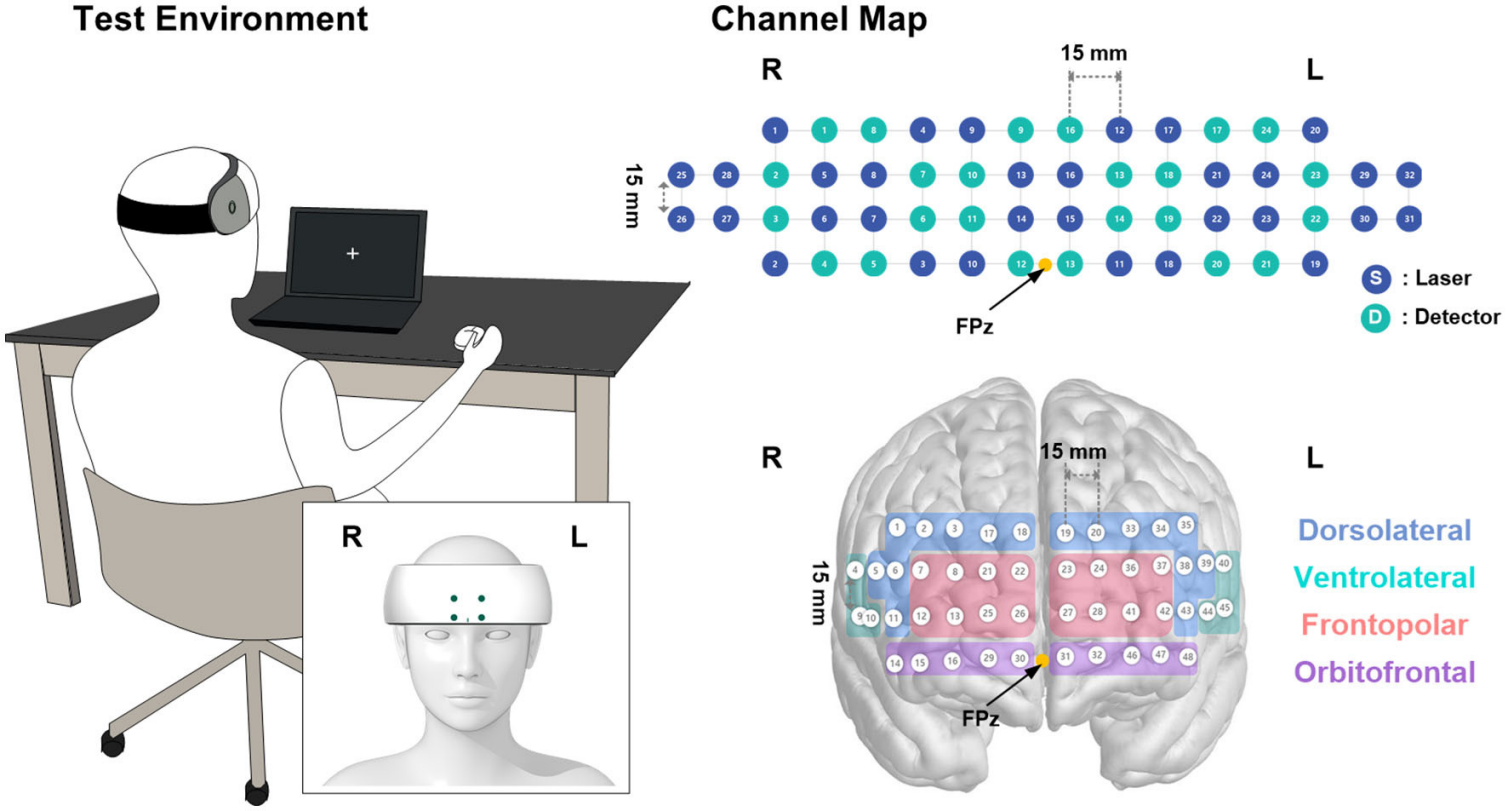
Visualization of the channel mapping of the device and test setup with the posture of the participant.

All tasks were performed in turn before (pre-treatment) and 1 h after (post-treatment) the dose treatment as described in Figure 2. During the 1-h consumption period after taking the dose treatment, the participants watched a non-arousing video (documentary showing the natural environment of a forest) so that the dose was completely absorbed. The time of testing was selected to coincide with the time of maximum pharmacodynamic effects, as observed in animals or human studies, of EGCG or GCG (Ullmann et al., 2004). To minimize the learning effect of all tasks at post-treatment, the duration of the pre-treatment tasks was one-third that of the post-treatment tasks, as shown in Figure 2. The participants were tested individually in a private room. They sat in a comfortable chair and were instructed to remain relaxed and only move their right hand, restraining any major body movements such as crossing or shaking their legs (Figure 3). The computerized battery of cognitive tasks comprised the following.

#### 2.4.1 Resting state

During the rest period, the participants engaged in a 3-min resting-state measurement, with their eyes open staring at a white-cross fixation presented on a screen with a black background. At the start of each resting state, a standard instruction screen informed the participant to stay calm, try not to move during the measurement, and relax. Moreover, it was emphasized that participants should not to fall asleep during the measurements.

#### 2.4.2 Mental arithmetic task

First, the participants were asked to rest for 30 s to obtain a pre-task baseline; then, they performed the control task for 30 s by repeating the five Korean vowels (“a,” “e,” “i,” “o,” “u”) aloud. Then, the participants performed the arithmetic task, which consisted of 10 sets of predefined multiplication questions in 60 s with four sets of double digits by single digit, four sets of single digits by double digit, and two sets of double digits by double digit questions sequentially. Meanwhile, the participants were asked to provide the answer to each question verbally. During the pre-treatment period, the arithmetic task was presented once (60 s). In the post-treatment period, the arithmetic tasks were presented three times (180 s total), with distinct predefined questions different from those in the pre-treatment period. Finally, a 10-s post-task resting period was measured.

#### 2.4.3 Corsi block-tapping task

The CBTT task consisted of nine gray cubes positioned in the original block formation of a Corsi board (Berch et al., 1998). The background color was black, and the target cubes were highlighted in blue for 500 ms each. During the memorization phase, target blocks were highlighted randomly depending on the block number (five to seven blocks, initially five blocks, and when the participant provided two consecutive correct answers, the number of blocks increased). After the presentation of all target squares was completed, the participants were asked to recall a sequence of five to seven squares in backward order, locate the blocks, and left click the target in backward order using a mouse. If they could not recall the sequence, they could pass the question. The participants were instructed to respond as quickly and accurately as possible. For each pre- and post-treatment period, the CBTT task was presented for 60 and 180 s, respectively. The CBTT task had pre- and post-task resting periods of 30 and 10 s, respectively.

#### 2.4.4 Verbal fluency task

First, the subjects were asked to rest for 30 s to obtain a pre-task baseline; then, they performed the control task for 30 s by repeating the five Korean vowels (“a,” “e,” “i,” “o,” “u”) aloud. Immediately after that, the participants were instructed to produce as many nouns as possible and speak out loud within the category provided on the screen for 30 s. The total number of nouns was counted by the operator, and voice was recorded during the task period for all participants for checking after the tasks were completed. For each pre- and post-treatment period, the control task and repetition of noun presentation were once (30 s) and three times (90 s), respectively. The provided category was “country” for pre-treatment task and the categories “animal, food, and sports” were used in a fixed order for post-treatment task. Finally, a 10-s resting period was measured for the post-task resting period.

### 2.5 Near-infrared spectroscopy and data processing

A portable fNIRS device (NIRSIT; OBELAB Inc., Seoul, Korea) was used to measure cerebral hemodynamic changes during the pre- and post-treatment periods. The fNIRS device measures hemodynamic variations using two wavelengths of near-infrared light (780 nm and 850 nm). A total of 48 channels with a 3-cm source-detector distance in the prefrontal cortex, consisting of 24 sources and 32 detectors, were monitored to trace the changes in optical intensity (Figure 3). The device was placed on each participant's forehead by aligning the center of the lowermost probes on the FPz, as defined by the international 10–20 electroencephalography system (Figure 3). The measurements were recorded at a sampling frequency of 8.13 Hz and stored on a tablet for data acquisition. Prior to the analysis, channels with insufficient signal integrity, that is, signal-to-noise ratio (SNR) < 30 dB, were excluded from further analysis. The measured signals were then filtered using a discrete cosine filter with a passband frequency of 0.01–0.1 Hz to eliminate high-frequency noise. Using the modified Beer–Lambert law, the relative concentration changes of oxy- (HbO_2_) and deoxy-hemoglobin (HbR) were monitored from the initial measurement. In this study, we mainly focused on the HbO_2_ concentration changes affected by GCG-GTE (Wightman et al., 2012).

A general linear model was fitted for each channel to derive a measure of the task-relevant fNIRS activation. For the “task” model, baseline periods immediately prior to each task (control task periods for MA and VFT, pre-task baseline for CBTT) and task period were included as regressors of interests together with temporal and dispersion derivatives of each regressor. Ten discrete cosine terms were included as nuisance regressors. An analogous model was fitted to the “rest” period. A first-level contrast between the baseline and task [-1 1] was used to define channels showing greater activation during the task period than that at baseline.

Considering the different coverage and size of the forehead across the subjects, the contrast beta from 48 channels was averaged, excluding those from rejected channels in order to maintain signal integrity, forming eight separate regions of interest (ROIs) that correspond to Brodmann areas such as the DLPFC and frontopolar, orbitofrontal, and ventrolateral prefrontal cortex (PFC), for further analysis (Figure 3). For details regarding Brodmann area grouping and estimated Montreal Neurological Institute coordinates of the channels, see OBELAB Inc. (2022). NIRSIT Channel Information, Seoul, Korea https://www.obelab.com/info/notice.php.

### 2.6 Statistical analysis

Before being submitted to further statistical tests, scores from the three cognitive tasks were summarized for a more straightforward conclusion. Performance (the number of correct answers for MA, proportion correct for CBTT, and number of words generated for VFT) in each task was z-scored within the entire dataset, collapsed across groups so that the task scores could be comparable to each other, and summarized to derive an overall cognitive performance measure by averaging the z-scores of the three tasks for each participant.

A series of 2 × 2 mixed analyses of variance (ANOVA), with group (GCG-GTE vs. placebo) as a between-variable and session (pre- vs. post-treatment) as a within-variable, were performed on physiological variables, cognitive performance measures, and fNIRS signals from Brodmann ROIs. In each ANOVA, a significant group-by-session interaction would indicate the effect of GCG-GTE on each dependent variable. Neither the main effect of group nor the main effect of session alone would be sufficient to support the potential effect of GCG-GTE on cognition or brain activation because the former would only indicate the difference (potentially, inherent difference by chance despite the random sampling) between the participants in the GCG-GTE and the placebo groups, regardless of pre- and post-treatment differences, and the latter would only indicate the difference between pre- and post-treatment sessions (potentially due to experience, either learning or fatigue), regardless of group assignment. Thus, only if a significant interaction between group and session factors is found, can we conclude that the result suggests that the group assignment (treatment) produced a difference between groups that had not been there during the pre-session or at least changed its pattern, thus demonstrating the effect of GCG-GTE.

All ANOVAs to test for the aforementioned interaction were performed with (thus, analysis of covariance in this case) the pre- and post-treatment cognitive task scores as covariates (with the exception of ANOVA on cognitive task scores themselves).

## 3 Results

### 3.1 Subject demographics and physiological data

After the participants were randomly assigned to either a placebo or GCG-GTE group and outliers were removed, 18 and 17 participants remained in the GCG-GTE and placebo groups, respectively. There was no difference in age [mean = 32.47, SD = 4.84, placebo; mean = 32.33, SD = 3.79 for GCG-GTE; *t*(33) = 0.094, *p* = 0.93] and sex [each group had eight females, χ^2^(1, *N* = 35) = 0.02, *p* = 0.88] between the two groups. No significant group-by-session interaction was found for heart rate [*F*(1, 31) = 0.03, *p* = 0.87] or blood pressure parameters [systolic: *F*(1, 31) = 0.42, *p* = 0.52; diastolic: *F*(1, 31) = 0.14, *p* = 0.71], indicating that GCG-GTE consumption did not cause significant changes in these physiological measures. The descriptive statistics and pairwise comparison results for the physiological measures in each session are summarized in Table 2.

**Table 2:** Mean and SD (in parentheses) of physiological measures of each group before and after treatment.

**Measurement**	**Session**	**Placebo**	**GCG-GTE**	**Group differences^†^**
Heart rate	Pre-treatment	74.82 (7.92)	75.72 (11.57)	*t* = −0.37, *p* = 1.0
	Post-treatment	70.65 (8.69)	71.29 (11.15)	*t* = −0.95, *p* = 1.0
	**Session differences** ^†^	*t* = 2.73, *p* = 0.06	***t*** **=** **3.06**, ***p*** **=** **0.03**	
Systolic blood pressure	Pre-treatment	113.59 (10.52)	108.94 (10.99)	*t* = 1.12, *p* = 1.0
	Post-treatment	114.24 (12.75)	111.56 (10.27)	*t* = 0.57, *p* = 1.0
	**Session differences** ^†^	*t* = −0.21, *p* = 1.0	*t* = −1.16, *p* = 1.0	
Diastolic blood pressure	Pre-treatment	70.88 (12.70)	72.33 (10.12)	*t = –*0.97, *p* = 1.0
	Post-treatment	73.06 (10.30)	75.22 (12.19)	*t = –*1.22, *p* = 1.0
	**Session differences** ^†^	*t = –*1.22 *p* = 1.0	*t* = −1.8, *p* = 0.49	

GCG-GTE, (–)-gallocatechin gallate-rich green tea extracts; SD, standard deviation.

^†^All direct comparisons (t-tests) are from *post-hoc* comparisons after mixed-effect ANOVAs with Group x Session effect; all p-values were adjusted with the Bonferroni correction for comparing a family of 6 pairwise comparisons. Results in bold are statistically significant.

### 3.2 Cognitive task performance

**Figure 4:**
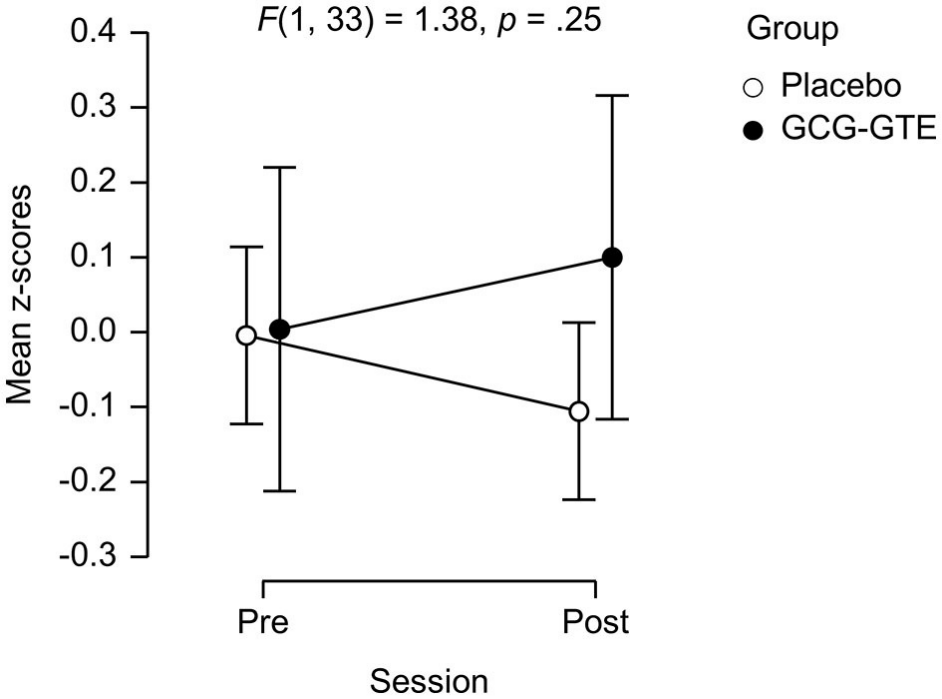
The performance on the cognitive tasks of each group (mean z-scores) during each session. Error bars indicate the 95% confidence interval.

The ANOVA on the combined “cognitive performance measure” yielded neither main effects nor the interaction between group and session [*F*(1, 33) = 1.38, *p* = 0.25]. However, the cognitive performance measures of the two groups were nearly identical during the pre-treatment session, but the performance of the GCG-GTE group slightly improved during the post-treatment session, whereas that of the placebo group slightly decreased, showing divergence between the two groups (Figure 4). Details of the scores for each task and the combined cognitive performance measures before and after the treatment in each group as well as statistics from pairwise comparisons are summarized in Table 3.

**Table 3:** Mean and SD (in parentheses) of behavioral performance of each group before and after treatment.

**Task**	**Session**	**Placebo**	**GCG-GTE**	**Group differences^†^**
Mental arithmetic (# of correct answers)	Pre-treatment	8.09 (1.62)	8.06 (1.12)	*t* = 0.03, *p* = 1.0
	Post-treatment	22.91 (3.95)	22.67 (4.59)	*t* = 0.23, *p* = 1.0
	**Session differences** ^†^	***t*** **=** **−18.45**, ***p*** ** < 0.001**	***t*** **=** **−18.71**, ***p*** ** < 0.001**	
Corsi block-tapping test (proportion correct)	Pre-treatment	0.56 (0.28)	0.40 (0.29)	*t* = 1.93, *p* = 0.35
	Post-treatment	0.33(0.12)	0.35 (0.23)	*t* = −0.15, *p* = 1.0
	**Session differences** ^†^	***t*** **=** **3.67**, ***p*** **=** **0.005**	*t* = 0.92, *p* = 1.0	
Verbal fluency test (# of words generated)	Pre-treatment	17.12 (3.14)	19.78 (5.33)	*t* = −1.44, *p* = 0.94
	Post-treatment	30.94 (6.04)	34.65 (6.64)	*t* = −2.17, *p* = 0.21
	**Session differences** ^†^	***t*** **=** **−10.31**, ***p*** ** < 0.001**	***t*** **=** **−11.64**, ***p*** ** < 0.001**	
Overall cognitive performance (*z*-score)	Pre-treatment	−0.004 (0.69)	0.004 (0.66)	*t* = −0.04, *p* = 1.0
	Post-treatment	−0.11 (0.61)	0.10 (61)	*t* = −0.95, *p* = 1.0
	**Session differences** ^†^	*t* = 0.84, *p* = 1.0.	*t* = −0.82, *p* = 1.0	

GCG-GTE, (–)-gallocatechin gallate-rich green tea extracts; SD, standard deviation.

^†^All direct comparisons (t-tests) are from *post-hoc* comparisons after mixed-effect ANOVAs with Group x Session effect; all p-values were adjusted with the Bonferroni correction for comparing a family of 6 pairwise comparisons. Results in bold are statistically significant.

### 3.3 Near-infrared spectroscopy: oxy-hemoglobin concentration change

**Figure 5:**
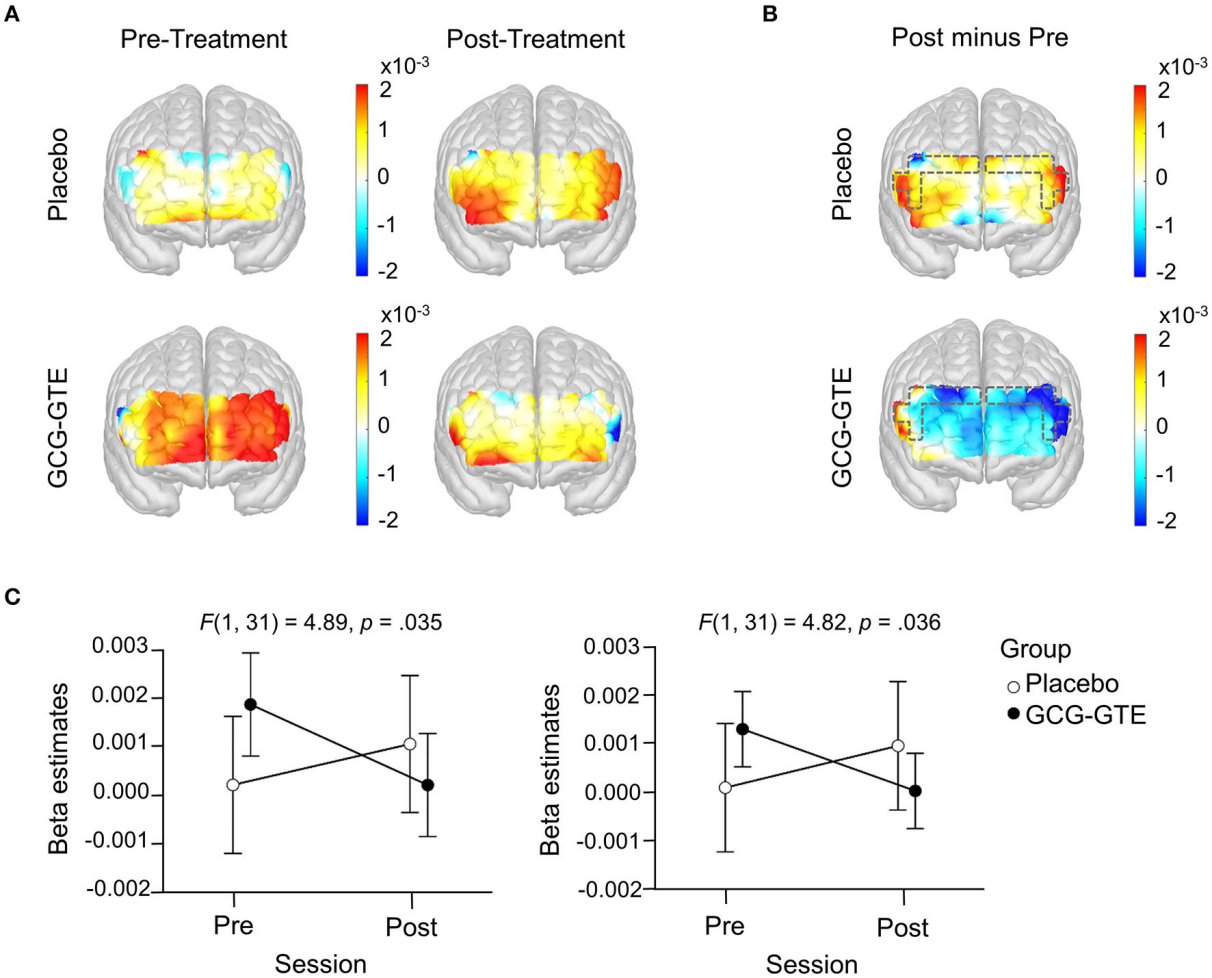
Generalized linear model beta activation maps for task period; **(A)** pre- and post-treatment of the green tea extract (GCG-GTE group) or placebo supplement (placebo group) during tasks. Regions with warmer colors show higher activation during cognitive tasks; **(B)** the difference between post- and pre- treatment session in the placebo group (upper panel) and in the GCG-GTE group (bottom panel). Regions with warmer colors demonstrate higher activation during post-session than pre-session whereas regions with cooler colors demonstrate higher activation during pre-session than post-session. The bilateral DLPFC ROIs (marked with dashed line) demonstrate a significant group x session interaction, resulting in reduced activation in the GCG-GTE group and increased activation in the placebo group after the respective treatments; **(C)** summary of the beta amplitude in the bilateral DLPFC ROIs demonstrating significant group x session cross-over interactions.

Task-related beta estimates from the bilateral DLPFC demonstrated a significant group-by-session interaction, indicating that GCG-GTE treatment changed the pattern of brain activation in these regions [*F*(1, 31) = 4.89, *p* = 0.035 and *F*(1, 31) = 4.82, *p* = 0.036, for the left and right DLPFC, respectively]. No main effects or *post-hoc* comparisons were found significant. Figure 5A shows the prefrontal responses during the pre- and post-treatment sessions in each group.

The amount of change before and after each treatment can be demonstrated by the contrast between post-treatment and pre-treatment activation. Figure 5B illustrates this contrast. As shown in the figure, the placebo group demonstrated higher activation during the post-session in most of the PFC regions, however, conversely, GCG-GTE group showed lower activation after treatment in most of the PFC regions.

Specifically, as shown in Figure 5C, the interactions found in these bilateral DLPFC regions were crossover, implying that activation size was greater in the GCG-GTE group than in the placebo group (although the difference was not statistically significant) during pre-treatment, but this pattern flipped during post-treatment. Figure 5A (2 × 2 activation maps) illustrates the overall prefrontal activation pattern of each group in each session. This demonstrates that the flip observed in the bilateral DLPFC is more or less universal across PFC regions. In other words, reduced activation in the GCG-GTE group after treatment and increased activation in the placebo group were found across the PFC regions, but this was most evident in the bilateral DLPFC ROIs, reaching statistical significance.

**Figure 6:**
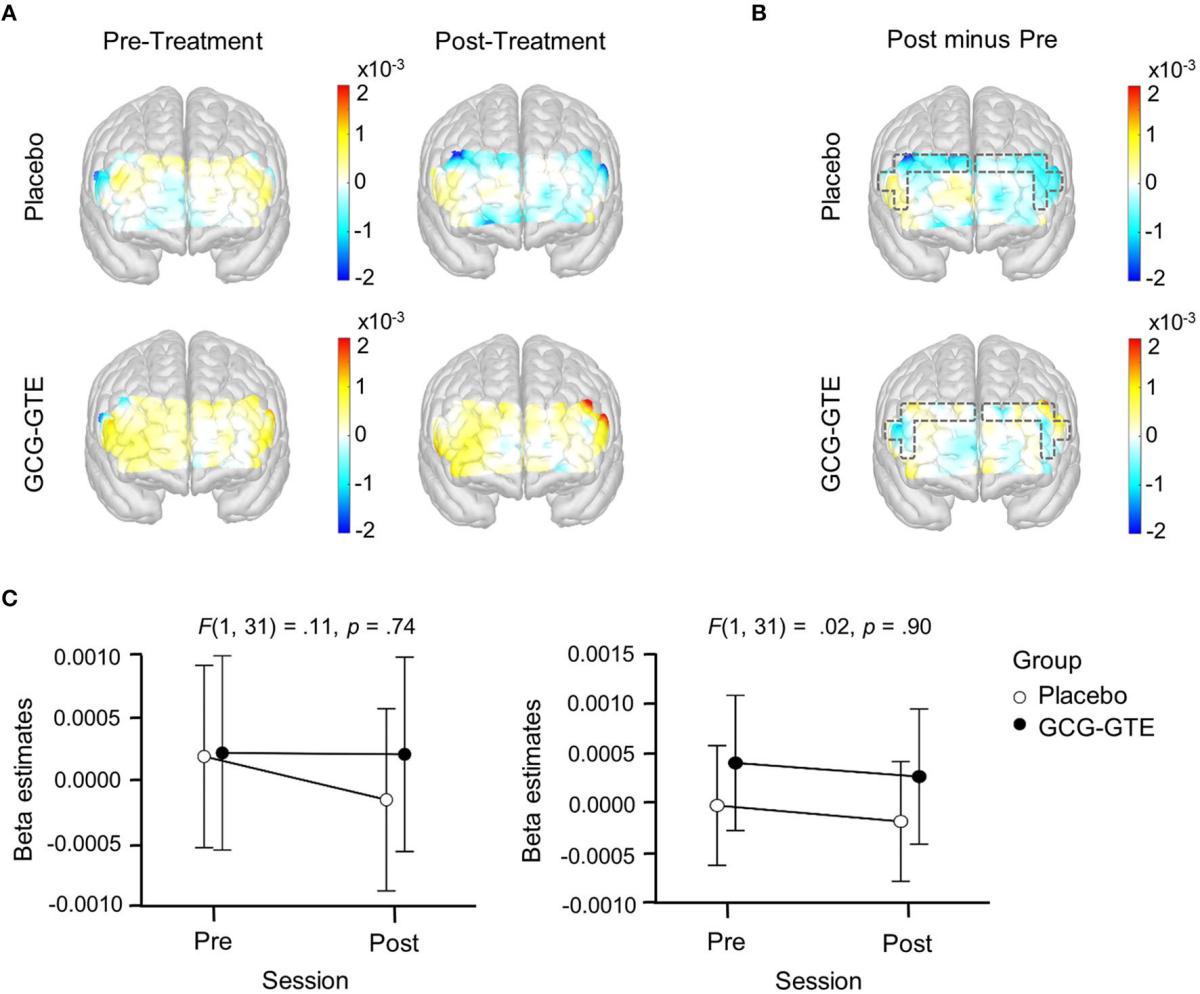
Generalized linear model beta activation maps for rest period; **(A)** pre- and post-treatment of the green tea extract (GCG-GTE group) or placebo supplement (placebo group) during rest. Regions with warmer colors show higher activation; **(B)** the difference between the post- and pre- treatment rest period in the placebo group (upper panel) and GCG-GTE group (bottom panel). Regions with warmer colors demonstrate higher activation during post- treatment rest than pre-treatment rest, whereas regions with cooler colors demonstrate higher activation during pre-treatment rest than post-treatment rest; **(C)** beta amplitude taken from the bilateral DLPFC ROIs where significant group x session cross-over interactions were found during task period was summarized for the purpose of comparison. During rest, neither the ROIs nor the other PFC regions demonstrated any significant group x session interactions.

Importantly, when the analogous ANOVA model was fitted to the data from the three-minute rest period, which was immediately followed by the task period, the same interaction was not found in any of the PFC regions (*p* > 0.05 for all), suggesting that the observed group × session interaction was a task-specific response to the treatment. Figure 6 summarizes the results of the rest-period analysis.

**Figure 7:**
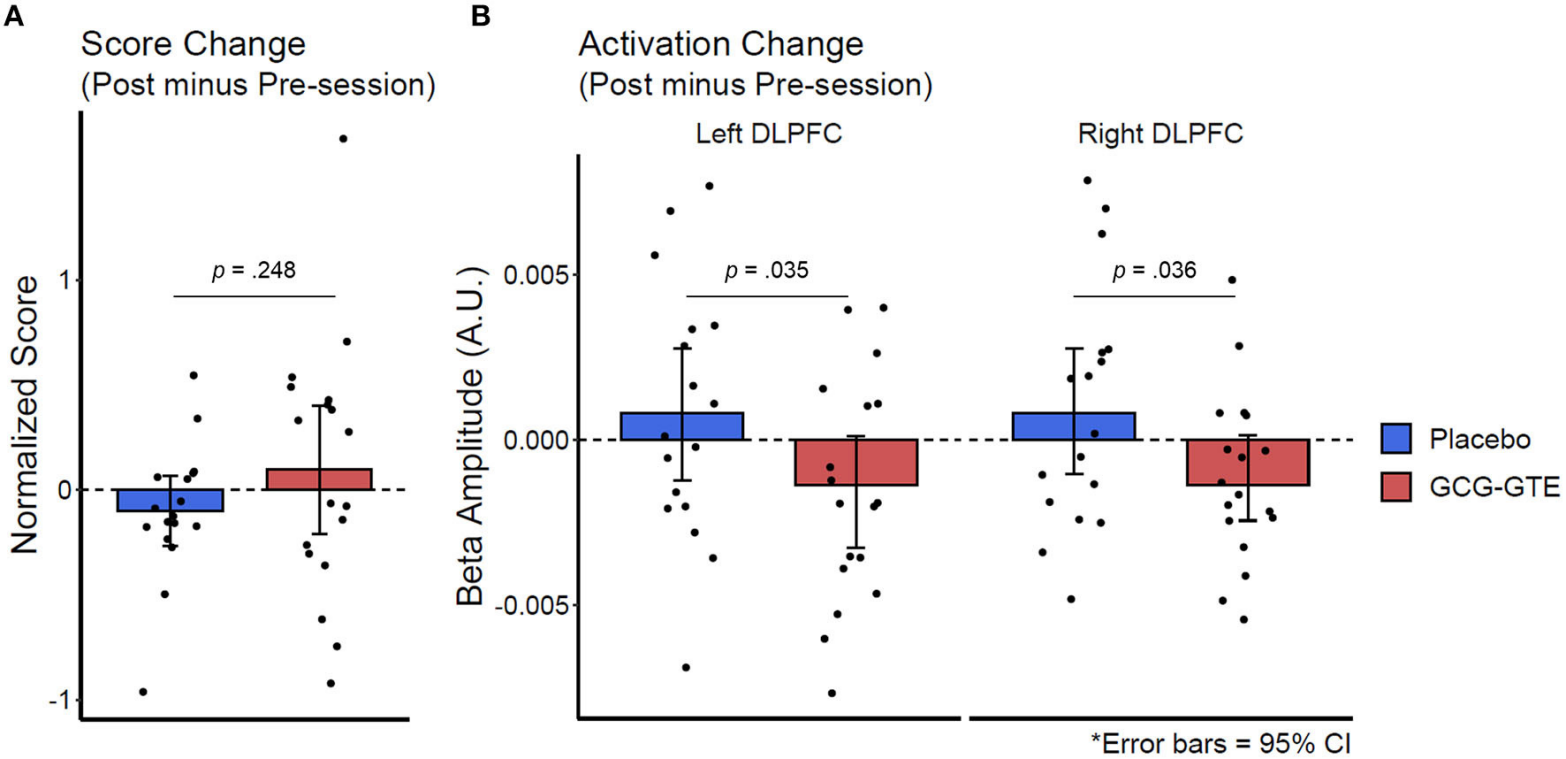
Change in behavioral performance score **(A)** and the bilateral DLPFC activation **(B)** after treatment. The changes were calculated by subtracting pre-treatment observations (baseline) from post-session observations.

Lastly, the aforementioned numerical improvement in behavioral performance and reduction in the bilateral DLPFC activation in the GCG-GTE group can be better demonstrated when the initial numerical differences between the groups during the pre-treatment session were corrected by calculating the magnitude of change between the pre- and post-session of each group using their pre-treatment observations as a baseline. Figure 7 juxtaposes the behavioral and neuronal changes after treatment, clearly illustrating that the two groups demonstrated changes in opposite directions. Table 4 lists the number of participants in each group who demonstrated a decrease or increase in behavioral performance, left DLPFC activation, and right DLPFC activation after treatment. The groups showed behavioral and neuronal changes in opposite directions. Specifically, the GCG-GTE group demonstrated better behavioral performance and lower DLPFC activation, whereas the placebo group demonstrated poorer behavioral performance and higher DLPFC activation after their respective treatments.

**Table 4:** Change in behavioral performance scores and left and right DLPFC activation.

**Group**	**Behavioral**		**Left DLPFC**		**Right DLPFC**	
	**Decreased**	**Increased**	**Decreased**	**Increased**	**Decreased**	**Increased**
Placebo	11 (64.7 %)	6 (35.3 %)	8 (47.1 %)	9 (52.9 %)	8 (47.1 %)	9 (52.9 %)
GCG-GTE	9 (50 %)	9 (50 %)	12 (66.7 %)	6 (33.3 %)	13 (72.2 %)	5 (27.8 %)
Total	20 (57.1 %)	15 (42.9 %)	20 (57.1 %)	15 (42.9 %)	21 (60 %)	14 (40 %)

The number in each cell indicates the number of participants in each group (and their percentage in parentheses), showing either an increasing or decreasing trend after treatment.

DLPFC, dorsal lateral prefrontal cortex; GCG-GTE, (–)-gallocatechin gallate-rich green tea extracts.

## 4 Discussion

This double-blind, placebo-controlled study investigated the acute effects of a single 450 mg dose of GCG-GTE on cognitive function and its accompanying cerebral hemodynamics. This study showed that a single dose of GCG-GTE tended to elicit improved cognitive performance compared with a placebo, although the difference was not statistically significant. In terms of cerebral hemodynamic modulation, the consumption of GCG-GTE led to a decrease in HbO_2_ concentration in the bilateral DLPFC during the task period relative to the consumption of placebo.

Given that changes in HbO_2_ are closely associated with task-related brain activity (Roy and Sherrington, 1890), the GCG-GTE-related task-dependent decrease in HbO_2_ indicates a decrease in neuronal activity in the bilateral DLPFC region. Critically, the observed decrease in bilateral DLPFC activation is unlikely to indicate a lack of necessary task-relevant neuronal computation, considering that behavioral performance outcomes were improved at the same time in the GCG-GTE group. This pattern of HbO_2_ reduction is consistent with the report by Wightman et al. (2012), who demonstrated that a single dose of EGCG resulted in reduced HbO_2_ in the frontal cortex during cognitive tasks.

Similar divergence between improved task performance and decreased HbO_2_ concentrations in the PFC has been reported in several studies on the effect of cognitive enhancers, also known as “nootropics,” on HbO_2_ measured via fNIRS (Ramasubbu et al., 2012; Best et al., 2021). In addition, Ramasubbu et al. (2012) reported that the administration of a single dose of methylphenidate, one of the most popular cognitive enhancers, induced a decrease in HbO_2_ concentration in the right lateral PFC, which was accompanied by improved performance in working memory tasks. Another nootropic, *Bacopa monnieri* extract, showed reduced activation of the PFC concomitant with improved cognitive task performance (Best et al., 2021).

Taken together, these results suggest that the GCG-GTE-induced decrease in DLPFC activation during task engagement may be interpreted as relief of the mental workload, thus, it is carefully speculated that GCG-GTE may act as a cognitive enhancer that enables similar or higher levels of cognitive performance with less mental fatigue (Froestl et al., 2012; Dunst et al., 2014; Urban and Gao, 2014; Suliman et al., 2016; Causse et al., 2017). The behavioral performance listed in Table 3 and the greater PFC activation observed in the placebo group during the post-treatment session support this speculation.

As the post-treatment session was three times longer than the pre-treatment session (3 min vs. 1 min for MA and CBTT, 1.5 min vs. 0.5 min, for VFT), it can be assumed that the participants might have experienced a potential increase in task-induced mental fatigue. Supporting this claim, Table 3 shows that the performance of both groups during the post-treatment session was lower than expected. That is, given the three times longer task duration, the number of correct answers in the MA task, as well as the number of words generated in the VFT in the post-treatment session, were expected to be three times that in the pre-treatment session. In addition, if mental fatigue or task difficulty were similar across sessions, the proportion of correct answers in the CBTT is expected to be equivalent across sessions. However, contrary to these expectations, this was not observed, suggesting that the task-induced fatigue or task difficulty in the post-session was potentially higher (and hence, may have induced greater PFC activation) than that in the pre-treatment session, which is exactly what the placebo group demonstrated. Again, considering these conditions, a decrease in PFC activation after GCG-GTE consumption can be attributed to the relief of mental workload. Whether it is based on improved neural efficiency (Ayaz et al., 2012, 2013; Causse et al., 2017; Curtin and Ayaz, 2019) or on the appropriate level of stress as Yerkes-Dodson law states (Teigen, 1994) after GCG-GTE consumption is unclear at this point and requires further investigation.

To the best of our knowledge, this is the first report of the acute cognitive and neural effects of GCG-GTE. By observing the treatment effect in the bilateral DLPFC, whose relation to high-level cognition is well-documented in the literature (MacDonald et al., 2000; Curtis and D'Esposito, 2003) and by demonstrating that the effect was only observed during the task, and not rest, the current study provides converging evidence regarding the cognitive benefits of GCG-GTE. Despite its merits, this study has several limitations. First, it did not draw a specific conclusion about the cognitive domain to which GCG-GTE might have brought the greatest benefit. Such a conclusion would have been difficult to ascertain, mostly because the pre-session performance of the two groups was not equivalent across the cognitive tasks. The within-subject averaging across tasks, described in Section 2.6, equated the pre-session performance so that the performance of the two groups during the post-session could be compared to each other. This averaging maneuver is justified because it is difficult to hypothesize that GCG-GTE will have specific potential benefits for any particular cognitive task or domain. Rather, it is more natural to assume that its potential cognitive benefit is universal across domains, and the tasks selected here are only a few examples that measure overall higher-level cognition.

In conclusion, our results suggests that green tea extract reduced bilateral DLPFC activation in the GCG-GTE group, whereas the placebo group showed increased activation during the post-treatment session. The greater activation during the post-treatment can be naturally attributed to increased task difficulty and task duration, which makes the observed reduction in the GCG-GTE group rather striking. Critically, this interaction was not observed during the rest period, when no recruitment of the DLPFC pertaining to cognitive processing was expected.

## Data availability statement

The raw data supporting the conclusions of this article will be made available by the authors, upon reasonable request.

## Ethics statement

Informed consent was obtained before the experiment according to the procedure approved by the Amorepacific R&I Institutional Review Board (IRB: 2019-1CR-N076S). This study was also registered with the Clinical Research Information Service (Registration Number: KCT0004808), Republic of Korea (URL link: https://cris.nih.go.kr/cris/index/index.do). The studies were conducted in accordance with the local legislation and institutional requirements. The participants provided their written informed consent to participate in this study.

## Author contributions

JC, H-SK, GK, S-YC, and J-MK contributed to conception and design of the study. GK, H-SK, and J-MK conducted clinical research. JC and J-MK organized the database and performed the statistical analysis. JC, S-YC, and H-SK wrote the first draft of the manuscript. All authors wrote sections of the manuscript, contributed to manuscript revision, read, and approved the submitted version.
